# MiR-196a Promotes Pancreatic Cancer Progression by Targeting Nuclear Factor Kappa-B-Inhibitor Alpha

**DOI:** 10.1371/journal.pone.0087897

**Published:** 2014-02-04

**Authors:** Fengting Huang, Jian Tang, Xiaohong Zhuang, Yanyan Zhuang, Wenjie Cheng, Wenbo Chen, Herui Yao, Shineng Zhang

**Affiliations:** 1 Department of Gastroenterology, Sun Yat-sen Memorial Hospital, Sun Yat-sen University, Guangzhou, China; 2 Department of Gastroenterology, The Sixth Affiliated Hospital, Sun Yat-sen University, Guangzhou, China; 3 Department of Oncology and Hematology, Hainan Provincial Nongken Hospital, Haikou, China; 4 Department of Medical Oncology, Sun Yat-sen Memorial Hospital, Sun Yat-sen University, Guangzhou, China; H. Lee Moffitt Cancer Center & Research Institute, United States of America

## Abstract

Aberrant expression of miR-196a has been frequently reported in different cancers including pancreatic cancer. However, its function in pancreatic cancer has not been fully elucidated. Here, we investigated the expression pattern and the biological role of miR-196a in pancreatic cancer cell lines, as well as its interaction with a metastasis-related gene, nuclear factor-kappa-B-inhibitor alpha (NFKBIA). We demonstrated that miR-196a was up-regulated in human pancreatic cancer cell lines compared with immortalized pancreatic ductal epithelial cells by means of microRNAs microarray and qRT-PCR. Furthermore, down-regulation of miR-196a in PANC-1 suppressed its proliferation and migration with an increase in G_0_/G_1_ transition and decreased expression of Cyclin D1 and CDK4/6. Meanwhile, an increased expression in E-cadherin and decreased expression in N-cadherin and Vimentin were also observed. We identified a novel miR-196a target, NFKBIA, and down-regulation of miR-196a enhanced the expression of NFKBIA protein. Luciferase assay confirmed that NFKBIA was a direct and specific target of miR-196a. Silencing NFKBIA in PANC-1 cells enhanced its proliferation and migration. Taken together, our findings indicate that miR-196a is highly expressed in pancreatic cancer cell lines, and may play a crucial role in pancreatic cancer proliferation and migration, possibly through its downstream target, NFKBIA. Thus, miR-196a may serve as a potential therapeutic target for pancreatic cancer.

## Introduction

Pancreatic cancer is an aggressive malignancy with one of the worst outcomes among all cancers. For all stages combined, the 5-year relative survival rate is only 5% [1]. The high mortality of pancreatic cancer could be partly due to the ability of pancreatic cancer cells to acquire invasive characteristics during the early stages of carcinogenesis. Thus, it is likely that even in the stage of an apparently localized disease, micrometastases may be already present in distant organ sites [2]. Conventional chemotherapy is rarely curative for metastatic pancreatic cancer. Treatment strategies that specifically target and prevent metastases might therefore have the potential to significantly improve the prognosis of this dismal disease.

Recent studies have shown that microRNAs (miRNAs) play a critical role in the regulation of various biological and pathologic processes, including metastasis [3]. These small, noncoding molecules exert their regulatory effects by binding to the 3′ untranslated region of target mRNA, causing either degradation of mRNA or inhibition of their translation to functional proteins. The expression of miRNAs has been recognized as integral components of many normal biological processes involving cell proliferation, differentiation, apoptosis, and stress resistance [4]. More importantly, it has been recently suggested that aberrant upregulation or downregulation of specific miRNAs and their targets in various types of cancer is associated with the development and progression of cancer [5]. The aberrant expression of some miRNAs has been shown to be involved in pancreatic cancer carcinogenesis [6], [7]. Moreover, miR-196a has been found to be overexpressed in pancreatic cancer, and significantly correlated with poor survival rate [8]. However, the mechanism of its function in pancreatic cancer remains unclear.

The nuclear factor κB (NF-κB) plays a significant role in the regulation of immune response [9] and inflammation [10]. It comprises a family of transcription factors involved in the regulation of a wide variety of biological process, and growing evidences demonstrated its involvement in tumorigenesis [11]–[14]. It has been implicated in many hallmarks of cancer development and progression, including growth factor-independent proliferation [15], inhibition of apoptosis [16], and tissue invasion and metastasis [17]. Also, emerging evidences imply that NF-κB activation plays an important role in the progression of pancreatic cancer [11], [18]–[20]. Inhibition of NF-κB sensitizes human pancreatic cancer cells to apoptosis [21]. NFKBIA, also known as IκBα, is one of the family members of cellular proteins that inhibit the NF-κB transcription factor. NFKBIA inhibits NF-κB by masking the nuclear localization signals (NLS) of NF-κB protein and keeping it sequestered in an inactive state in the cytoplasm [22]. In addition, NFKBIA blocks the ability of NF-κB to bind to DNA, which is essential for the function of NF-κB [23]. It has been shown that there is an enrichment of specific single-nucleotide polymorphisms and haplotypes of NFKBIA in Hodgkin's lymphoma, colorectal cancer and multiple myeloma, suggests that NFKBIA might be a tumor suppressor [24]–[26].

In this study, we demonstrate that miR-196a is overexpressed in pancreatic cancer cell lines and have investigated the effect of down-regulation of miR-196a on a pancreatic cancer cell line, PANC-1. We have elucidated that NFKBIA is a target of miR196a, and miR-196a plays an important role in the development and progression of pancreatic cancer likely by targeting NFKBIA.

## Materials and Methods

### Cell lines

Four human pancreatic cancer cell lines PANC-1, Capan-2, BxPC-3 and SW1990 were purchased from the Chinese Academy of Sciences (Shanghai, P.R. China), and an immortalized pancreatic ductal epithelial cell line H6C7 was kindly provided by Prof. Ming-sound Tsao (Ontario Cancer Institute, Toronto University, Canada), and was incubated in this study as reported previously [27]. Four human pancreatic cancer cell lines (Chinese Academy of Sciences, Shanghai, P.R. China) were cultured in DMEM (Gibco, Grand Island, NY) supplemented with 10% fetal bovine serum (FBS, HyClone, Logan, UT), 100 unites/ml penicillin G, and 100 µg/ml streptomycin. H6C7, obtained from Prof.Ming-sound Tsao of Ontario Cancer Institute (Ontario, Canada), was cultured at 37°C in keratinocyte serum free medium (K-SFM) (Invitrogen, Carlsbad, CA, USA) containing 100 u/ml penicillin, 100 u/ml streptomycin, 0.2 ng/ml recombinant endothelial growth factor (rEGF) and 20 ng/ml Bovine Pituitary Extract (BPE). In all experiments, cells were maintained at 37°C in a humidified 5% CO_2_ air atmosphere.

### GeneChip Microarray of miRNAs

The miRNA gene expression profile of four human pancreatic cancer cell lines and H6C7 was determined by GeneChip microarray analysis (Affymetrix, Santa Clare, CA, USA). Synthesis of cDNA, hybridization to chips, and washes were performed according to the manufacture's protocol. GeneChips were scanned at 3 mm density with a GeneArray Scanner (Affymetrix). Images were inspected to ensure that all chips had low background but bright hybridization signals. Mean fluorescence signal intensity for each probe was quartile normalized. The average of three mean signals for each miRNA probe was normalized to that for an added control oligonucleotide and was log_2_ transformed. Each miRNA probe was assessed for expression based on a Wilcoxon Rank-Sum test of the miRNA probe set signals compared to the distribution of signals from the background. The Student's *t*-test was used to determine significant differences in miRNA expression between human pancreatic cancer cell line and the immortalized pancreatic ductal epithelial cell line H6C7, where *P*<0.05 was interpreted as significant.

### Quantitative real-time RT-PCR (qRT-PCR)

To analyse the expression of miR-196a, qRT-PCR was performed in Four human pancreatic cancer cell lines (PANC-1, Capan-2, BxPC-3 and SW1990) and an immortalized pancreatic ductal epithelial cell line H6C7. Briefly, total RNA was extracted from the cells using TRIZOL (Invitrogen, CA, USA) according to the manufacturer's instruction. U6 was validated as the normalizer. Total RNA was reversely transcribed using the corresponding RT Primer and the TaqMan MicroRNA Reverse Transcription Kit (Applied Biosystems, Foster City, CA, USA). The PCR primer for mature miR-196a was designed as follow: miR-196a sense, 5′-GCT CTG GCT CCG TGT CTT CAC TCC C-3′, reverse, 5′-TGC CCC AGC ACA GCC CCC GTC CCT C-3′. The expression of miR-196a and its control U6 were detected using TaqMan miRNA assay system (Applied Biosystems, Foster City, CA, USA).

### Transfection of PANC-1 cells

Two pairs of synthetic, chemically modified short single- or double-stranded RNA oligonucleotides: anti-miR-196a and its appropriate negative control (anti-miR-NC), miR-196a micmics and its appropriate negative control (miR-NC) were purchased from GenePharma (Shanghai, P.R. China). NFKBIA-siRNA (si-NFKBIA) and its appropriate negative control (si-NC) were purchased from GeneChem (Shanghai, P.R. China). Transfection was performed by Lipofectamine 2000 reagent (Invitrogen, Carlsbad, CA, USA) according to the manufacturer's instructions. For transfection, 2×10^5^ PANC-1 cells were seeded into each well of a 6-well plate and incubated overnight. The expression levels were quantified 24 h after transfection.

### Cell proliferation assay

Cell proliferation was detected by WST-8 method. Anti-miR-196atransfected PANC-1 cells and anti-miR-NCtransfected PANC-1 cells were harvested and dissociated into single cell suspension, 1.2×10^3^ cells were seeded in 96-well plate per well. Also, si-NFKBIA transfected PANC-1 cells, si-NC transfected PANC-1 cells, si-NFKBIA+anti-miR-196a transfected PANC-1 cells and si-NC+anti-NC PANC-1 cells were harvested and dissociated into single cell suspension, 2×10^3^ cells were seeded in 96-well plate per well. Cell proliferation was examined at different hours (24 h, 48 h, 72 h). WST-8 reagent (10 µl per well) from Cell Counting Kit-8 (Dojindo, Kumamoto, Japan) was added, incubated for 4 h, and absorbance was determined with a multiwell spectrophotometer (BioTek, VT, USA) at 450 nm and 630 nm.

### Cell migration assay

Cell migration assay was carried out by using Transwell chamber (Corning, New York, USA) with pore size of 8.0 µm. 72 h after transfection, total 10^5^ cells were resuspended in serum-free media and seeded in the upper compartment of the chamber. The lower compartment was loaded with full culture media containing 10%FBS. After being incubated at 37°C for 8 hour, the chamber was fixed, 0.1% crystal violet-stained and counted.

### Flow cytometry analysis

To detect the effect of down-regulation of miR-196a on cell cycle and apoptosis, flow cytometry analysis was performed. For cell cycle analysis, anti-miR-196a-transfected PANC-1cells were harvested at different hours (24 h, 48 h, 72 h) after transfection, and were trypsinized and fixed with ice-cold 70% ethanol for 18 h at 4°C. The fixed cells were stained with 50 mg/mL propidium iodide (BD Pharmingen, San Diego, CA) and 50 mg/mL RNase and then analysed using a flow cytometer (BD Pharmingen, San Diego, CA). For apoptosis analysis, anti-miR-196a-transfected PANC-1cells were also harvested at different hours (24 h, 48 h, 72 h) after transfection, stained with FITC-Annexin V and Propidium iodide (PI) and then analysed using a flow cytometer (BD Pharmingen, San Diego, CA). Anti-miR-NC-transfected PANC-1cells were performed as control.

### Immunofluorescence analysis

To investigate the phenotype changes of PANC-1 transfected with anti-miR-196a, immunofluorescence analysis was performed. Observation of morphology of Blank, anti-miR-NC and anti-miR-196a group was performed by microscope. The expression of E-cadherin and vimentin, markers of EMT, were detected by immunofluorescence on 3^rd^ day after transfection. The cells were washed with PBS and fixed in 4% paraform for 15 min on ice. After two more phosphate-buffered solution(PBS) wash, the cells were covered with 0.5% Triton 100 for 15 min on ice, then washed with PBS and incubated with 5% non-fat milk for 1 h at room temperature to block nonspecific binding of IgG. The cells were incubated with primary antibody mouse anti-human E-cadherin (Abcam, MA, USA) or vimentin (Abcam) for 2 h at room temperature, then washed with PBS and incubated with fluorochrome–conjugated secondary antibody at room temperature for 30 min in a dark chamber. The cells were washed with PBS and covered with DAPI to stain the nuclei. We took random photos using 200-fold magnification.

### Western blot analysis

The concentration of total protein extracted from Blank, anti-miR-NC and anti-miR-196a group was determined with a BCA Protein Assay kit (Pierce, USA). Equal amounts of protein were separated by 10% SDS-PAGE and electrophoretically transferred to PVDF membranes (Millipore, Bedford, MA, USA) using a mini trans-blot. Mouse anti-human Cyclin D1 (Abcam), CDK4 (Abcam), CDK6 (Abcam), E-cadherin (Abcam), Vimentin (Abcam), Rabbit anti-human N-cadherin (Cell Signaling Technology, MA, USA), NFKBIA (Santa Cruz Biotechnology, Santa Cruz, CA, USA), were used to detect the expression of homologous proteins. GAPDH (Santa Cruz Biotechnology) was used as an internal control. Electrochemiluminescence was performed with a Chemilmager 5500 imaging system (San Leandro, CA, USA), according to the manufacturer's instructions.

### 3′UTR luciferase reporter assay

The human NFKBIA 3′UTR luciferase reporter construct (NFKBIA-3′UTR WT) was generated by cloning NFKBIA mRNA 3′UTR sequence into downstream of pMIR-Report construct (land, Guangzhou, P.R. China). The miR-196a target site-mutation NFKBIA 3′UTR luciferase reporter (NFKBIA-3′UTR mutation) construct was generated by employing direct-site mutagenesis using mutation primers which mutate the miR-196a binding site from ACTACCT to ATCGATC. PANC-1 cells were co-transfected with miR-196a plasmid and wild-type or mutant NFKBIA 3′UTR luciferase reporter construct and luciferase activities were measured using the Dual-Glo Luciferase. Data were normalized by dividing Firefly luciferase activity with that of Renilla luciferase.

### Statistical analysis

Data are presented as mean ± standard deviation (SD), computed using the SPSS software, version 13.0. The means were then compared using a one-way ANOVA with LSD among groups or student *t* test between groups. *P*<0.05 indicated statistical significance.

## Results

### MiR-196a is overexpressed in pancreatic cancer cell lines

**Figure 1:**
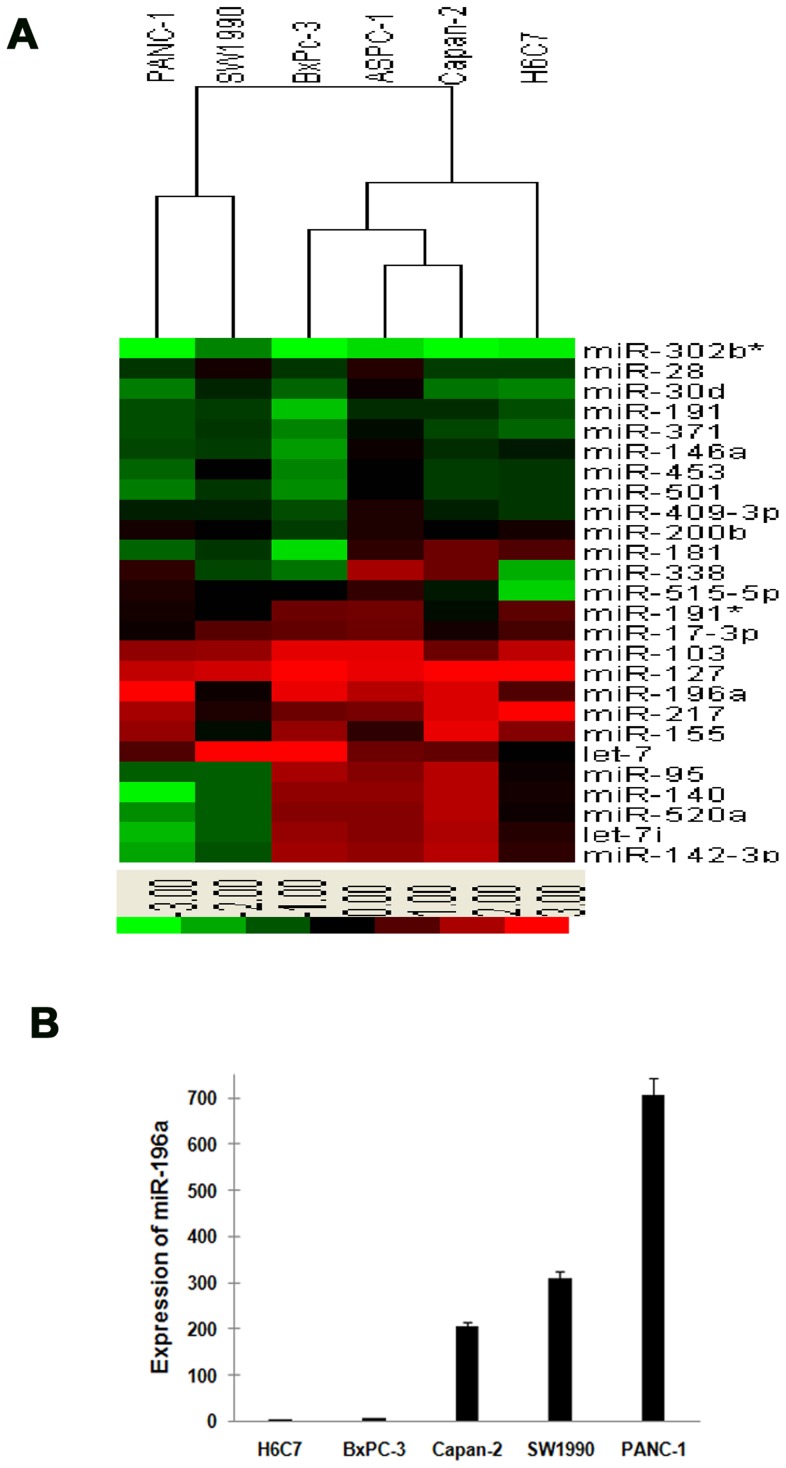
MiR-196a was overexpressed in pancreatic cancer cell lines. (A) Hierarchical clustering analysis of miRNAs that were either differentially up- or downregulated in pancreatic cancer cell lines and H6C7. MiRNAs that scored a differential value of 1 or greater were categorized as differentially upregulated, and miRNAs that scored a value of −1 or less were categorized as differentially downregulated. The scale bar across the bottom of the heatmap depicts SD change from the mean. MiR-196a expression was significantly higher in pancreatic cancer cell lines in microarray. (B) Validation of miR-196a expression level in pancreatic cancer cell lines by qRT-PCR. MiR-196a was significantly upregulated in pancreatic cancer cell lines. Expression of miR-196a was (706.4±9.4)-fold in PANC-1 cells, (310.1±7.5)-fold in SW1990 cells, (7.6±1.1)-fold in BxPC-3 cells and (204.9±4.8)-fold in Capan-2 cells, compared with H6C7 (*, *P*<0.05).

To explore the role of miR-196a in pancreatic cancer development, firstly we examined the expression of miR-196a in four pancreatic cancer cell lines (Capan-2, BxPC-3, PANC-1 and SW1990) and immortalized pancreatic ductal epithelial cell line H6C7 by miRNAs microarray and real-time RT-PCR. The hierarchical cluster revealed that miR-196a expressions in pancreatic cancer cell lines were much higher than that in H6C7 (Figure 1A). Meanwhile, the result of real-time RT-PCR was in accordance with microarray. Expression of miR-196a was (706.4±9.4)-fold in PANC-1 cells, (310.1±7.5)-fold in SW1990 cells, (7.6±1.1)-fold in BxPC-3 cells and (204.9±4.8)-fold in Capan-2 cells, compared with H6C7 (*P*<0.05) (Figure 1B). It is implied that miR-196a may play a role in the development of human pancreatic cancer.

### Effect of miR-196a on proliferation and apoptosis of PANC-1 cells

**Figure 2:**
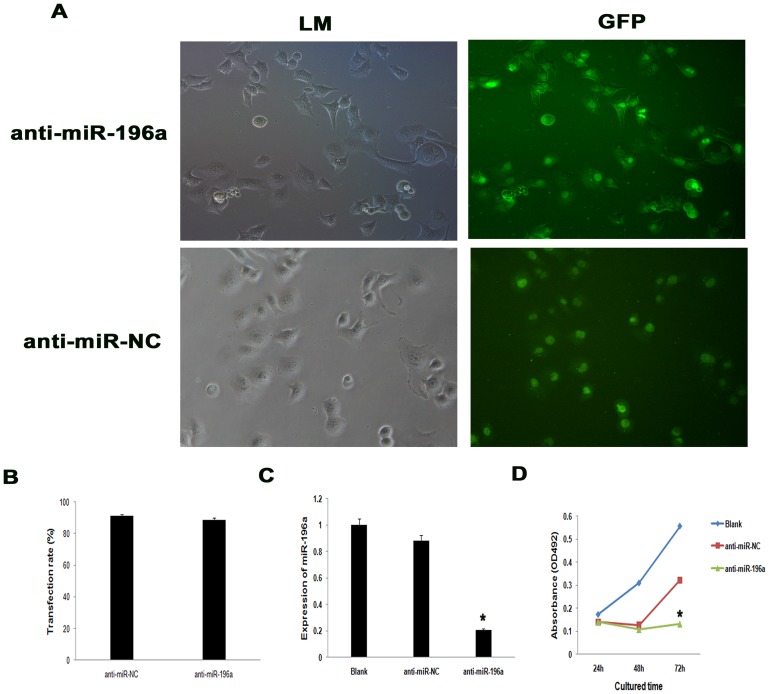
Downregulation of miR-196a inhibited proliferation of pancreatic cancer cell line PANC-1. (A) Transfection of anti-miR-196a and anti-miR-NC in PANC-1. (B) Comparision of transfection rate between anti-miR-196a and anti-miR-NC in PANC-1. The transfection rate of anti-miR-196a was (88.76±2.25)%, while the transfection rate of anti-miR-NC was (91.09±1.77)% (*P*>0.05). (C) Expression of miR-196a by qRT-PCR. (D) Growth curve among anti-miR-196a, anti-miR-NC and parental PANC-1 cells. Cell proliferation was significantly reduced in anti-miR-196a group compared with that of anti-miR-NC group at 72 h (*, *P<0.05*). *LM*: light microscope.

As shown in Figure 1, miR-196a expression was much higher in pancreatic cancer cell lines compared with the immortalized pancreatic ductal epithelial cell line, especially in PANC-1. To further assess the biological role of miR-196a in pancreatic cancer, we chose PANC-1 for the follow experiments for its high expression of miR-196a and investigated the effect of targeted knockdown of miR-196a on cell proliferation and apoptosis. It was revealed that anti-miR-196a was efficiently introduced into the cells (Figure 2A, Figure 2B) and down-regulated miR-196a expression level (Figure 2C). WST-8 assay revealed that the cell proliferation was significantly impaired in PANC-1 cells transfected with anti-miR-196a at 72 h compared with control group anti-miR-NC (*P*<0.05) (Figure 2D), while alteration of miR-196a expression had no significant effect on cell proliferation compared with control group anti-miR-NC at 24 h (*P* = 0.987) and 48 h (*P* = 0.241).

**Figure 3:**
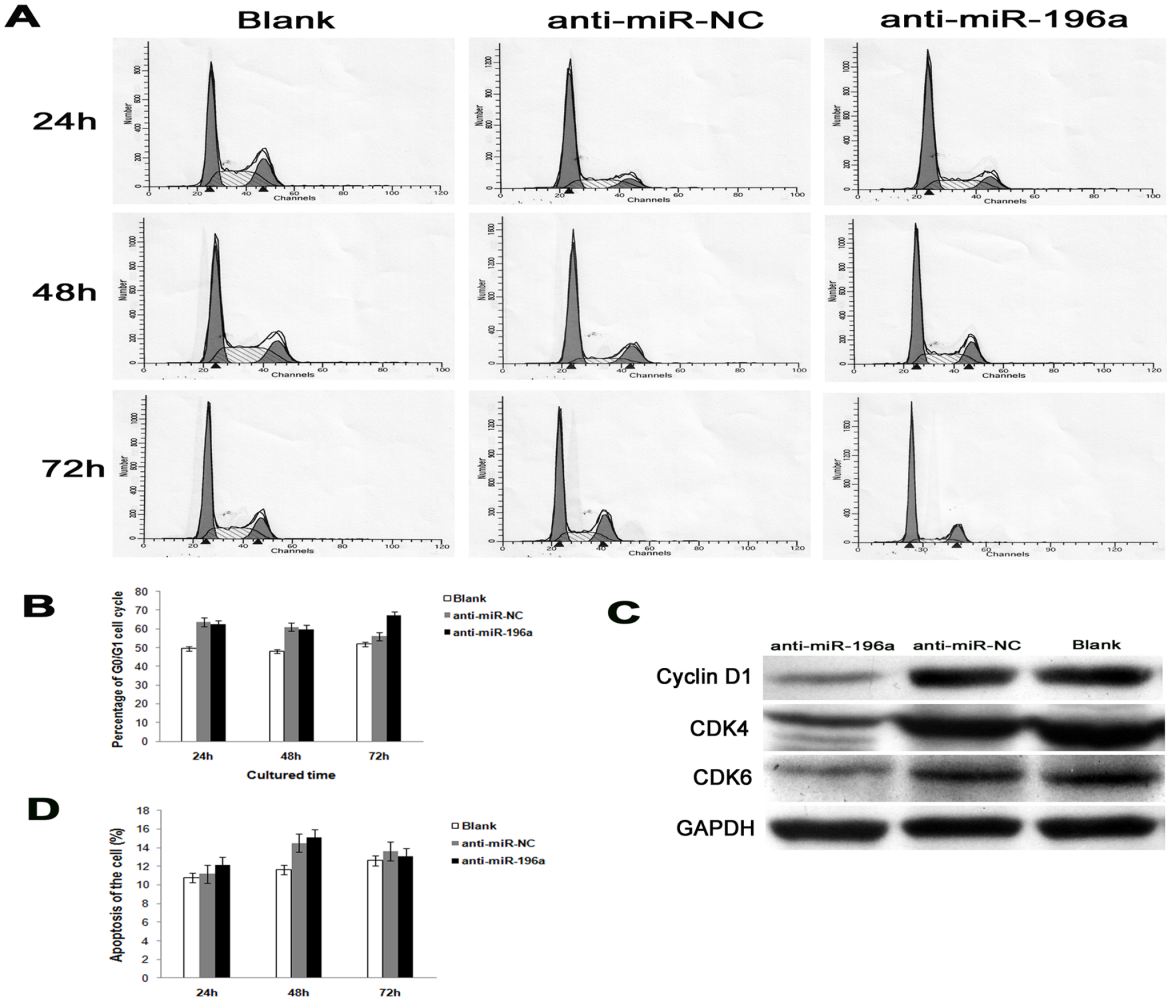
Repression of miR-196a increased G_0_/G_1_ phase but had no effect on apoptosis of PANC-1 cells. (A) Representative flow cytometry analysis of cell cycle in PANC-1 with and withdrawl silencing miR-196a at 24 h, 48 h and 72 h. (B) Comparison of cell cycle among anti-miR-196a, anti-miR-NC and Blank. The percentage of cells at G_0_/G_1_ phase at 72 h was increased from (56.07±7.93)% (anti-miR-NC) to (67.20±3.12)% (anti-miR-196a) (*, *P*<0.05), while there was no statistic significance in G_0_/G_1_between anti-miR-196a group and anti-miR-NC group at 24 h and 48 h. (C) Representative western blot analysis showed downregulation of Cyclin D1 and CDK4/6 expression after suppression of miR-196a in PANC-1 cells at 72 h. (D) Comparison of apoptosis among anti-miR-196a, anti-miR-NC and Blank.

Further, we determined whether cell cycle or apoptosis would contribute to the inhibition of proliferation. Flow-cytometric analysis was carried out. After silencing miR-196a by anti-miR-196a at 72 h, percentage of G_0_/G_1_ was significantly increased compared with control group anti-miR-NC, (67.20±3.12)% (anti-miR-196a) vs (56.07±7.93)% (anti-miR-NC) (*P*<0.05), while there was no statistic significance in G_0_/G_1_between anti-miR-196a group and anti-miR-NC group at 24 h (*P* = 0.825) and 48 h (*P* = 0.785) (Figure 3A, Figure 3B). Moreover, we detected the expression of Cyclin D1 and CDK4/6 protein. It was interesting that decreased expressions of Cyclin D1 and CDK4/6 were observed after silencing miR-196a (Figure 3C). Meanwhile, there was no significant difference of apoptosis among Blank, miR-NC and anti-miR-196a group in PANC-1 cells (Figure 3D). Taken together, the results indicate that knockdown of miR-196a suppresses cell proliferation, partly due to G_0_/G_1_ arrest with Cyclin D1 and CDK4/6 expression decreased, but it is not associated with induction of apoptosis.

### Downregulation of miR-196a suppresses PANC-1 cell migration

**Figure 4:**
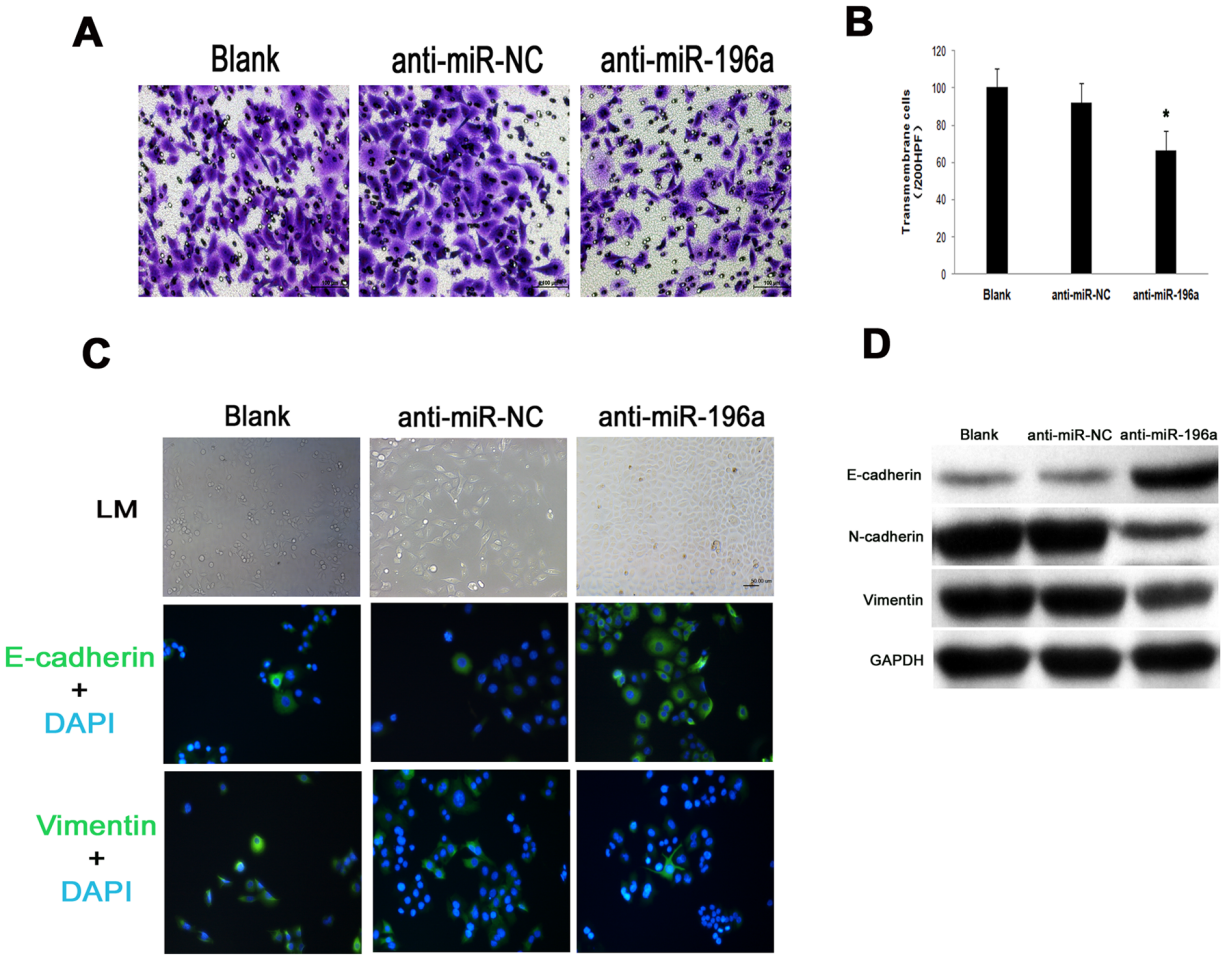
Downregulation of miR-196a suppressed PANC-1 cell migration. (A) Representative transwell assay indicated that the migration ability of PANC-1 cells was markedly reduced by down-regulation of miR-196a. (B) Comparison of transmembrane cells among anti-miR-196a, anti-miR-NC and Blank (*, *P*<0.05). (C) Morphological changes and immunofluorescence staining of MET markers among anti-miR-196a, anti-miR-NC and blank. (D) Representative western blot analysis revealed that mesenchymal-epithelial transition contributed to suppression of PANC-1 cell migration after silencing miR-196a. After silencing miR-196a, E-cadherin expression increased, as well as expression of N-cadherin and Vimentin decreased.

To investigate whether miR-196a had an effect on facilitating pancreatic cancer cell migration, we performed Transwell assay using PANC-1 cells. PANC-1 cell was chosen because of its overexpression of miR-196a and imitation of pancreatic cancer biology better than other cell lines, especially in cell migration analysis [28]. Transwell assay revealed that the migration ability of PANC-1 cells was markedly reduced by down-regulation of miR-196a, approximately 28% compared with control (*P*<0.05) (Figure 4A, Figure 4B). Meanwhile, we wondered whether mesenchymal-epithelial transition (MET) contributed to suppression of PANC-1 cell migration after silencing miR-196a, we first observed the morphology of PANC-1 before and after transfection with anti-miR-196a. To our interest, the cell morphology changed remarkably after transfection. In blank and anti-miR-NC group, some cells were partly spindle shape, whereas, anti-miR-196a cells were tightly bound, polygon cells with an epithelial phenotype. Meanwhile, we detected MET markers (vimentin and E-cadherin) expression by immunofluorescence. Increased expression of E-cadherin was observed after silencing miR-196a, with expression of vimentin decreased (Figure 4C). Further, we examined the protein expression associated with MET. Strikingly, with the reduction of PANC-1 cell migration after silencing miR-196a, increased expression of E-cadherin protein was observed, as well as decreased expression of N-cadherin and vimentin (Figure 4D). These results indicate that miR-196a indeed contributes to the migratory phenotype of pancreatic cancer cells, partly through MET.

### NFKBIA is a target of miR-196a in pancreatic cancer

**Figure 5:**
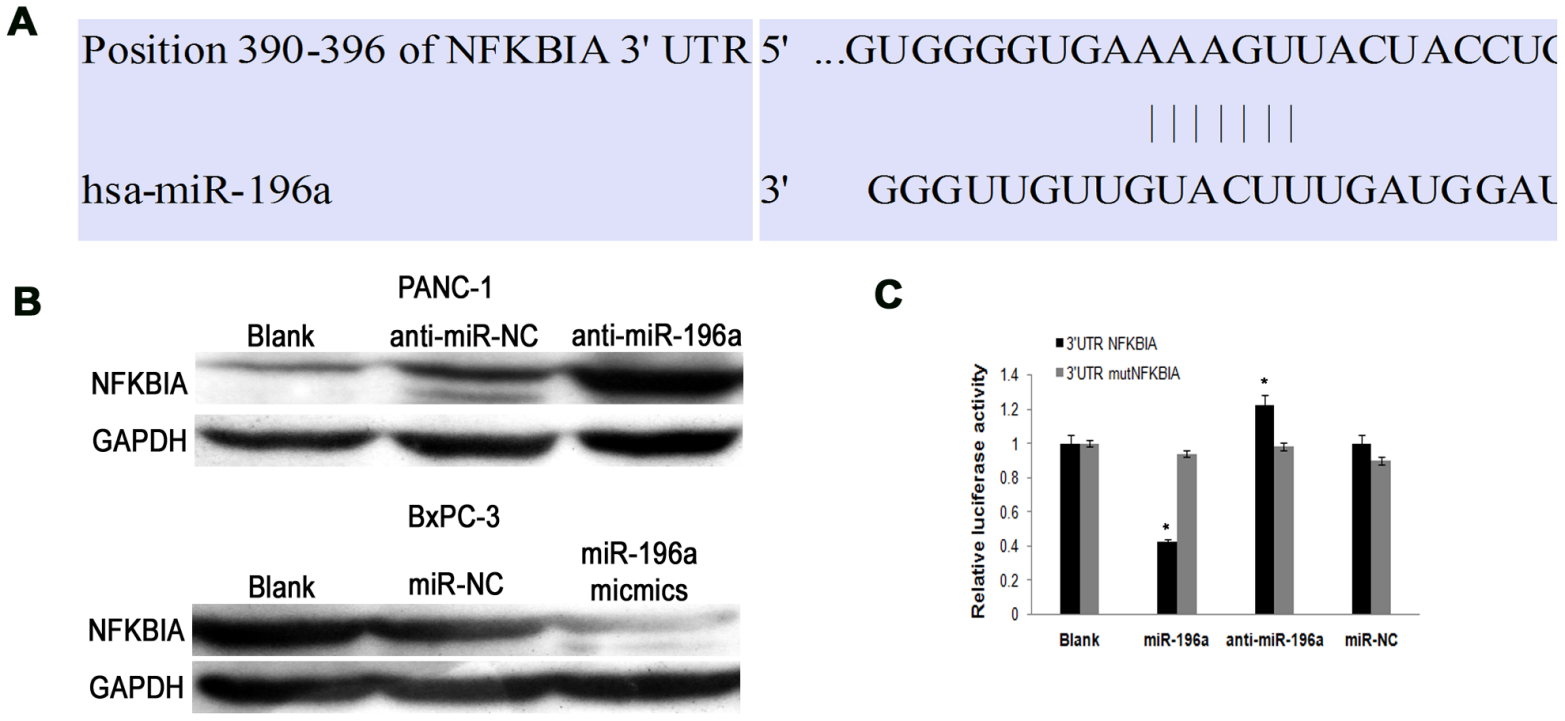
MiR-196a directly targets NFKBIA gene. (A) NFKBIA is a potential target gene of miR-196a predicted by computational analysis. (B) Representative western blot analysis showed the relationship between miR-196a expression and endogenous NFKBIA protein level. The inhibition of miR-196a in PANC-1 cells increased endogenous NFKBIA protein level, whereas overexpression of miR-196a in BxPC-3 cells attenuated endogenous NFKBIA protein level. (C) Insertion of NFKBIA3′UTR target sequences in a luciferase reporter vector leaded to diminished luciferase activity in presence of miR-196a in PANC-1 cells 24 h after co-transfection. Histograms showed the values resulting as the average ± SD from three independent co-transfections (*, *P*<0.05).

We then investigated the molecular mechanisms by which miR-196a regulates the migratory phenotype. The possible miR-196a target genes by database analysis are summarized in Table S1. Online search for miR-196a targeting genes by TargetScan, miRanda and PicTar revealed that NFKBIA, a proto-oncogene associated with migration and invasion, could be a potential target of miR196a (Figure 5A). We next determined whether NFKBIA expression was negatively associated with miR-196a level in pancreatic cancer cell lines. As proven in Figure 1, the expression of miR-196a was the highest in PANC-1 cells, and the lowest in BxPC-3 cells. Thus, we chose the two cell lines for further NFKBIA protein expression. To our interest, NFKBIA protein expression was higher in BxPC-3 cells than that in PANC-1 cells. Moreover, NFKBIA increased after down-regulation of miR-196a in PANC-1 cells, and decreased after up-regulation of miR-196a in BxPC-3 cells (Figure 5B). To ascertain direct miRNA-target interaction, we set up a luciferase reporter assay. As shown in Figure 5C, the luciferase activity in PANC-1 cells was decreased with WT construct by down-regulation of miR-196a level, which could be partly restored with mutant constructs. These results suggest that 3′UTR of NFKBIA is a direct target of miR-196a.

### Silencing NFKBIA promotes proliferation and migration of PANC-1 cells

**Figure 6:**
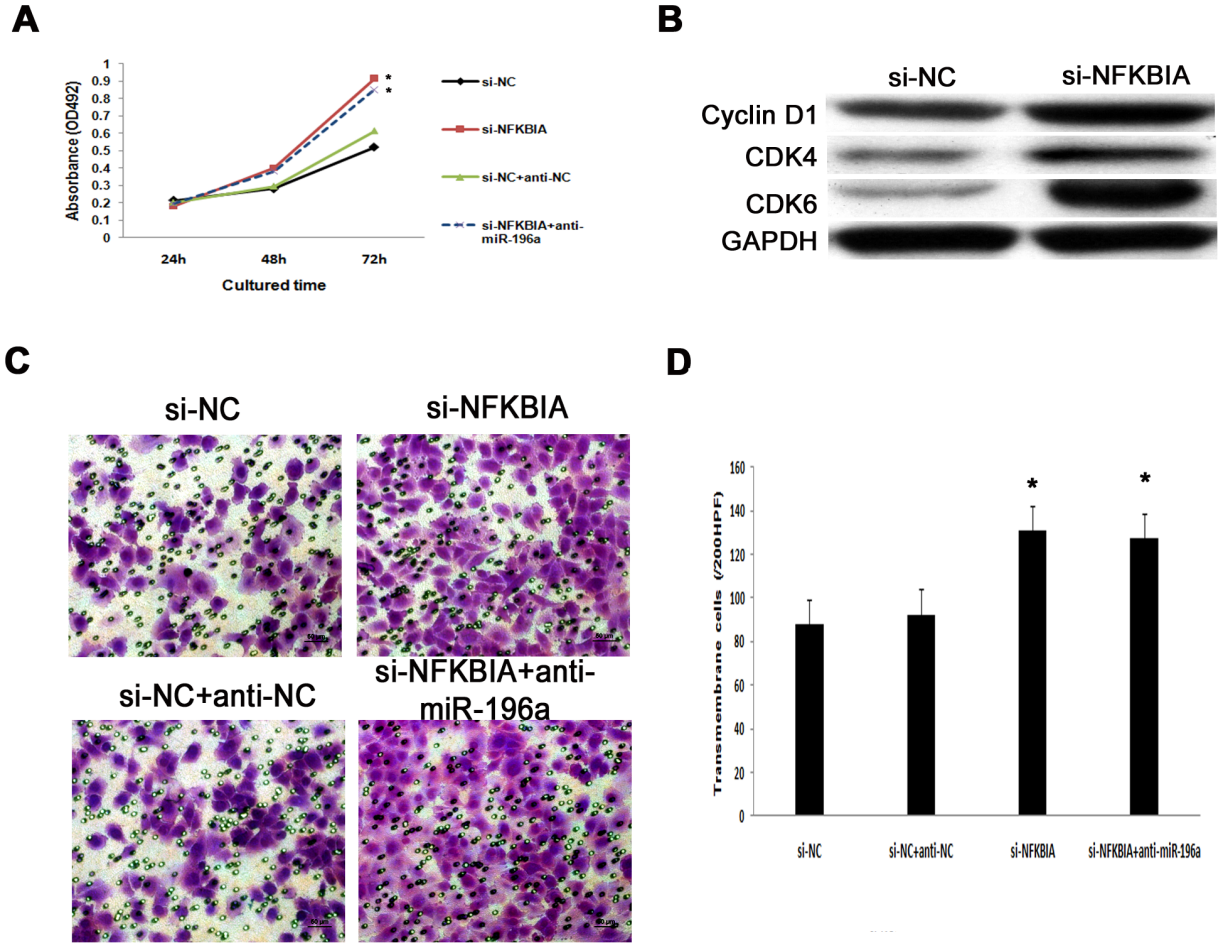
Effect of NFKBIA on PANC-1 cell proliferation and migration. (A) Growth curve among si-NFKBIA, si-NFKBIA+anti-miR-196a and their appropriate controls. WST-8 assay showed inhibition of NFKBIA enhanced PANC-1 cells proliferation (*, *P*<0.05). Meanwhile, dual inhibition of NFKBIA and miR-196a promoted cells proliferation. (B) Representative western blot analysis showed up-regulation of Cyclin D1 and CDK4/6 expression after suppression of NFKBIA in PANC-1 cells at 72 h. (C) Representative transwell assay indicated that the migration ability of PANC-1 cells was markedly attenuated by down-regulation of NFKBIA. Moreover, inhibition of NFKBIA blocked the effect of anti-miR-196a on cell migration. (D) Comparison of transmembrane cells among si-NFKBIA, si-NC, si-NFKBIA+anti-miR-196a, and si-NC+anti-miR-NC (*, *P*<0.05).

To further determine the biological role of NFKBIA in pancreatic cancer, we investigated the effect of targeted knockdown of NFKBIA in PANC-1 cells. Meanwhile, as proven before, NFKBIA is a target of miR-196a, in order to abrogate the effect of anti-miR-196a, we co-transfected siNFKBIA and anti-miR-196a in PANC-1 cells. We performed WST-8 assay to detect the cell proliferation. It was revealed that the cell proliferation was significantly increased in PANC-1 cells transfected with si-NFKBIA at 72 h compared with its control group si-NC (*P*<0.05), meanwhile, the cell proliferation was significantly increased in PANC-1 cells transfected with si-NFKBIA+anti-miR-196a at 72 h compared with its control group si-NC+anti-NC (*P*<0.05). There was of no statistic significance between si-NFKBIA and si-NFKBIA+anti-miR-196a (*P*>0.05) (Figure 6A). As shown before, expressions of Cyclin D1 and CDK4/6 decreased after silencing miR-196a. We further investigated the protein expressions of Cyclin D1 and CDK4/6 after silencing NFKBIA. To our interest, increased expressions of Cyclin D1 and CDK4/6 were observed after silencing NFKBIA (Figure 6B). Next, transwell assay suggested inhibition of NFKBIA promoted cells migration. Moreover, dual inhibition of NFKBIA and miR-196a promoted cells migration (Figure 6C, Figure 6D), which implied that inhibition of NFKBIA blocked the effect of anti-miR-196a on cell migration. These data suggest that inhibition of NFKBIA promotes pancreatic cancer cell promotion and migration.

## Discussion

MicroRNA profiling studies indicate that miR-196a is over-expressed in several cancers, such as breast cancer [29], colorectal cancer [30], gastric cancer [31], and pancreatic cancer [6], [7]. Interestingly, an increasing number of reports indicate that miR-196a plays important roles in development and progression of cancer. Overexpression of miR-196a is associated with high-risk grade, metastasis and poor survival among gastrointestinal stromal tumors [32]. MiR-196a has been found to promote proliferation and invasion of non-small cell lung cancer cell, indicating its important biological role in tumor progression [33]. It is reported that miR-196a is identified with increased expression to correctly differentiate pancreatic cancer from benign pancreatic tissue, and high expression of miR-196a is found to predict poor survival [6]. Meanwhile, It is reported that serum miR-196a expression levels in unresectable pancreatic cancer (stages III and IV) patients are significantly higher than those in resectable (stages I and II) patients [7]. Furthermore, serum miR-196a expression level is found to have a potential value in predicting median survival time of pancreatic cancer patients. In our research, we elucidated that miR-196a was over-expressed in pancreatic cancer and its up-regulation was significantly associated with migration potential, which may promote pancreatic cancer progression and lead to poor prognosis. EMT is believed to be an essential step for cancer invasion and metastasis [34], [35]. With decreased migration potential after silencing miR-196a, elevated expression of E-cadherin and decreased expression of N-cadherin and Vimentin were observed, which implied that mesenchymal-epithelial transition contributed to suppression of PANC-1 cell migration after silencing miR-196a. Furthermore, we demonstrated that miR-196a promoted pancreatic cancer proliferation through G_0_/G_1_ arrest and decreased Cyclin D1 expression and CDK4/6 expression but not apoptosis.

Moreover, we investigated the molecular mechanism of miR-196a in pancreatic cancer tumorigenesis. Emerging evidences imply that miR-196a contributes to tumor pathogenesis via the targeting of specific genes [36]-[39]. Our data showed that miR-196a contributed to proliferative and migratory potential of pancreatic cancer, which promoted our investigation on target genes associated with proliferation and migration. Nuclear factor-kappa B (NF-κB), a hallmark of the inflammatory response, is activated frequently in tumors and may play a crucial role in linking inflammation to tumor development and progression [40]. Previous studies demonstrated that NF-κB suppression in cancer inhibits cell proliferation, causes cell-cycle arrest, suggesting that NF-κB may play an important role in cell proliferation. Meanwhile, NF-κB inhibitor-α (NFKBIA), which represses NF-κB, is associated with distal metastasis of oral squamous cell cancer [41] and glioblastoma [42]. These findings prompted the hypothesis that NFKBIA might be a target gene of miR-196a. And it is demonstrated in our bioinformatic and the luciferase reporter data. Furthermore, silencing NFKBIA promotes pancreatic cancer cells PANC-1 proliferation and migration, consistent with the results of ectopic miR-196a expression in the same cells.

In conclusion, our results suggest that miR-196a is overexpressed in pancreatic cancer cell lines, and down-regulation of miR-196a by anti-miR-196a suppresses proliferation and migration of pancreatic cancer, partially by targeting NFKBIA.

## Supporting Information

Table S1 **Possible target genes of miR-196a.** Online search for miR-196a targeting genes by TargetScan, miRanda and PicTar revealed that NFKBIA could be a potential target of miR196a.
(DOCX)
