# Supplementary material for: MiR-196a Promotes Pancreatic Cancer Progression by Targeting Nuclear Factor Kappa-B-Inhibitor Alpha
Source: PLoS One. 2014 Feb 4;9(2):e87897. doi: 10.1371/journal.pone.0087897 (PMC3913664; doi:10.1371/journal.pone.0087897)
Supplement: Table S1 — Possible target genes of miR-196a. Online search for miR-196a targeting genes by TargetScan, miRanda and PicTar revealed that NFKBIA could be a potential target of miR196a. (DOCX) [file pone.0087897.s001.docx]

**Table S1.**

Possible target gene of miR-196a.

HOXC8 homeobox C8

MAP3K1 mitogen-activated protein kinase kinase kinase 1

IGF 1 insulin-like growth factor 1

FOXO1 forkhead box O1

USP 31 ubiquitin specific peptidase 31

HMGA2 high mobility group AT-hook 2

TGFβR III transforming growth factor, beta receptor III

C11orf57 chromosome 11 open reading frame 57

GATA6 GATA binding protein 6

RASSF 3 Ras association domain family member 3

NFKBIA nuclear factor-kappa-B-inhibitor alpha

SMAD6 SMAD family member 6

NAP1L1 nucleosome assembly protein 1-like 1

ZMYND11 zinc finger, MYND-type containing 11

E2F7 E2F transcription factor 7

LIN28A lin-28 homolog A (C. elegans)

COL1A1 collagen, type I, alpha 1

SCRT1 scratch homolog 1, zinc finger protein (Drosophila)

TMEM143 transmembrane protein 143
